# Clinical Epigenetics of Neuroendocrine Tumors: The Road Ahead

**DOI:** 10.3389/fendo.2020.604341

**Published:** 2020-12-15

**Authors:** Annamaria Colao, Filomena de Nigris, Roberta Modica, Claudio Napoli

**Affiliations:** ^1^ Department of Clinical Medicine and Surgery, Unesco Chair Health Education and Sustainable Development, Federico II University of Naples, Naples, Italy; ^2^ Department of Precision Medicine, University of Campania “Luigi Vanvitelli”, Naples, Italy; ^3^ Department of Clinical Medicine and Surgery, Federico II University of Naples, Naples, Italy; ^4^ Department of Advanced Medical and Surgical Sciences, University of Campania “Luigi Vanvitelli”, Naples, Italy

**Keywords:** epigenetics, neuroendocrine neoplasms, trials, biomarkers, neuroendocrine, neuroendocrine tumor

## Abstract

Neuroendocrine tumors, or NETs, are cancer originating in neuroendocrine cells. They are mostly found in the gastrointestinal tract or lungs. Functional NETs are characterized by signs and symptoms caused by the oversecretion of hormones and other substances, but most NETs are non-functioning and diagnosis in advanced stages is common. Thus, novel diagnostic and therapeutic strategies are warranted. Epigenetics may contribute to refining the diagnosis, as well as to identify targeted therapy interfering with epigenetic-sensitive pathways. The goal of this review was to discuss the recent advancement in the epigenetic characterization of NETs highlighting their role in clinical findings.

## Introduction

Neuroendocrine neoplasms (NENs) are a heterogeneous group of malignancies originating from neuroendocrine cells diffuse throughout the body. The gastroenteropancreatic (GEP) tract and the bronchopulmonary system represent the main site of origin. NENs are mostly sporadic, but in 10–30% they can arise within the context of familial syndromes, mainly multiple endocrine neoplasia type 1 (MEN1) (1). Incidence and prevalence of NENs have markedly increased in the last decades, irrespective of stage and grade (2). Clinical presentation and prognosis of NENs may widely vary. NENs can be functional when they release biologically active hormones that cause distinct clinical syndromes or more often may be non-functional, thus diagnosed incidentally or due to mass effect. Delayed diagnosis is common, as well as the detection of metastases, mainly to the liver, already at diagnosis. Patients with localized disease have a better prognosis, with 5-year survival ranging from 78 to 93%, while in metastatic disease, the 5-year survival is worse (19–38%), although improved over the past years (3). The improvement of survival rates may be the consequence of the availability of effective therapies, as well as earlier and more accurate clinical and pathologic diagnoses with relative downstaging. NENs have usually an indolent course and patients need life-long therapy. Notably, the landscape of the therapeutic options in NENs has considerably expanded in the last decades. The current systemic therapies for locally advanced or metastatic NENs include somatostatin analogs (SSAs), molecular targeted therapy with mTOR inhibitors (Everolimus), or anti-angiogenesis (Sunitinib), peptide receptor radionuclide therapy (PRRT) with either 90Yttrium (90Y) or 177Lutetium (177Lu) and chemotherapies with temozolomide, capecitabine or platinum-based regimens. These options can be used in sequence or association with surgery, locoregional treatments (e.g., radiofrequency ablation, cryoablation, chemoembolization, and radioembolization), and/or other drugs used as supportive therapies (e.g., telotristat, diazoxide and proton pump inhibitors) (4, 5). In this review we will focus on well or moderately differentiated neuroendocrine tumors (NETs), excluding neuroendocrine carcinomas (NEC) for their peculiar pathology and treatment.

## Epigenetic Modifications and Neuroendocrine Tumors

Epigenetic changes, such as DNA methylation and histone modification, are critical for regulating genes and non-coding RNA expression. Genomic alterations and gene mutations which are involved in the pathogenesis NETs, as MEN1, VHL-hypoxia-inducible factor, RASSF1A, have a consequence on the aberrant placement of epigenetic markers and related pathways (6–10).

Epigenetic mechanisms can modify gene expression altering DNA methylation status, histones post trascriptional modifications, and influencing the expression of non-coding RNAs. Hypermethylation of a promoter is a mechanism that determined gene silencing, while hypomethylation can lead to chromosomal instability and consequently influences gene expression (9, 10). Histone modifications involves the addition of methyl, acetyl, phosphorylation at different aminoacid residues of histone proteins. These modifications alter chromatin accessibility to transcription factors and lastly gene expression. MicroRNAs (miRNAs) and long noncoding RNAs are other layers of epigenetic regulation. They are small, or long sequences of non-coding RNAs regulating gene expression post-transcriptionally, considered to be a cancer-associated epigenetic mechanism (11).

## Methylation Patterns Relevance in the Pathogenesis of NETs and Clinical Findings

The pathogenesis of NETs is further to be elucidated, as in most other solid tumors. Nevertheless, epigenetic studies have improved our knowledge. Pancreatic neuroendocrine tumors (PNETs) account for 1 to 2% of all pancreatic tumors and most of them are sporadic and non-functioning, 5–7% arise within inherited syndromes, including MEN1, Von-Hippel Lindau (VHL) syndrome, neurofibromatosis type 1 (NF1), and tuberous sclerosis. The majority of familial PNETs are caused by germline inactivating mutations in the MEN1 gene, suggesting a key role in PNETs tumorigenesis. MEN1 gene encodes the transcription factor MENIN, ubiquitously expressed, and involved in many biological functions. MENIN, plays an essential role in chromatin remodeling and gene expression recruiting the H3K4me3 histone methyltransferase on mixed-lineage leukemia (MLL1) complex, regulating the expression of the cyclin-dependent kinase inhibitors, and influenced the epigenetic regulation of several genes (12). MEN1 mutations or loss of function deregulated cell growth in 75% cases of PNETs favoring hypermethylation of several tumor suppressor genes including RASSF1A (13), HIC-1, MLH1, CDKN2A, and MGMT (6, 7). Characteristics of the sporadic form of PNETs are mainly gene mutations in DAXX (death-domain-associated protein) or ATRX (alpha thalassemia/mental retardation syndrome X-linked) (12). Both DAXX and ATRX are chromatin remodellers and are involved in the incorporation of the histone variant H3.3 at the telomeres and pericentric heterochromatin necessary (14). Proteins loss, as well as mutations in DAXX or ATRX, are associated with chromosome instability (CIN), reduced genomic H3K9me, and aggressive PNET phenotype (12, 15). Increased risk of PNET was also associated with loss of chromosome 11q containing the genes Men1, but also DNA repair pathway genes as BRCA2 and ATM, and amplification region activating PIK3CA and mTOR pathway. In some cases associated with MENIN loss were also found mutation affecting VHL tumor suppressor gene that determined a constitutive hypoxia transcription factors (HIF) activation and uncontrolled angiogenesis (16, 17), suggesting that MENIN loss or mutation is a key initiator in PNET tumorigenesis (15, 18–21). In pulmonary NET in addition to MEN1 mutations affected also as histone lysine methyltransferase (SETD1B2, SETDB1), histone acetylation modifiers (BRWD3 and HDAC5) and ATP-dependent chromatin remodeling SMARCA1 indicating a key pathogenic role (22). Genomic profile of small intestinal NET (SI-NET) identify two different groups, one characterized by loss of chromosome 18, and another one characterized by the presence of chromosome 18 but with clustered gains on chromosomes 4, 5, 7, 14, and 20 (23). Correlation of loss of chromosome 18 and RASSF1A promoter hyper-methylation and hypo-methylation of long intergenic element 1 (LINE1) and ALU sequences was found in SI-NETs (24) although not associated with grade and tumor size (25).

In hereditary SI-NET causative role was attributed to germline mutations in IPMK (inositol polyphosphate multikinase) p53 activity and MutY DNA glycosylase genes (26) affecting the oxidative pathway. Above mentioned studies emerged that in SI-NET epigenetic machinery is not causative however the uncontrolled pathways of oxidative stress and genomic rearrangement activated several epigenetic modifications (27).

## Methylation Patterns Relevance as Differential Diagnostic Biomarkers

The DNA methylation profile of sporadic PNET, VHL and MEN1-related PNETs, and pancreatic islets were analyzed by Illumina array (850k array) with the goal to find novel diagnostic markers. The study identified a distinct cluster of methylation genes associated with VHL, sporadic and MEN1-related PNETs, indicated that mutations in these genes influence the epigenetic pathway and clinical presentation of diseases (28, 29). Differential methylation patterns were also reported among GEP-NETs (24, 30). Indeed, the analysis conducted in 60 tumors selected a pool of 807 genes. These gene sets were able to distinguish NETs in functional GEP-NETs (insulinoma, gastrinoma) and, non-function subtypes underlying the clinical and histological characteristics. Gastrinomas showed hypomethylation of genes including metalloproteinases (MMP1, MMP3, TIMP2, TIMP3), the serpin family (SERPINA5, SERPINB5), and oncogenes (IL2, MCF2, and MOS), whereas hypermethylation was reported for tumor suppressors (SMARCB1, CASP8, and NBL1) (24, 25, 30). Promoter hypermethylation of the IGF2 pathway was characteristic of insulinomas shedding a light on signaling responsible for their differentiation from a common origin (31). A study on SI-NET identified TCEB3C gene hypermethylation to be specific for this histology. Interestingly, treatment of SI-NET cell lines with the de-methylating agent decitabine and the histone methyltransferase inhibitor 3-deazaneaplaoncin A-induced TCEB3C re-expression, confirming an epigenetic regulation of this gene (32). Followed this stem study, Verdugo et al. and then Karpathakis et al. identified hyper-methylation of the gastric inhibitory polypeptide receptor (GIPR) as another specific marker of SI-NETs and reported hyper-methylation in several genes. They selected on chromosome 18 as laminin alpha 3 (LAMA3), serpin peptidase inhibitor clade B member 5 (SERPINB5), and factor receptor superfamily member 11a NFKB activator (RANK or TNSFRSF11A), suggesting that epigenetic silencing could be the possible second step in tumor development upon chromosome 18 loss (33–35).

## Methylation Patterns: Relevance in Prognosis and Response to Therapy

Since epigenetic changes play a key role in the progression of PNETs the finding to select an epigenetic prognostic factor, is crucial (34). In particular, some epigenetic changes area correlated to DAXX or ATRX protein loss because this complex regulates H3K9me and influenced DNA methylase. Indeed, promoter hypermethylation of RASSF1A and p57Kip2 in PNENs was responsible for NAP1L1 overexpression associated with the metastatic phenotype (35–38). Additionally, a peculiar group of PNETs named (CIMP) showed hypermethylation of CpG islands including tumor suppressor genes, such as RASSF1A, hMLH1, and hypomethylation of LINE-1 sequence. These peculiar epigenetic pathways were associated with poor prognosis and advanced stage of PNETs (39). While hypermethylation ofCDKN2A was associated with early tumor recurrence and poor outcomes of GEP-NETs (40). A general decrease in methylation levels was observed in SI-NET metastases compared to the primary tumors. In particular, differential methylation of AXL, CRMP1, FGF5, CXXC5, and APOBEC3C genes were detected in primary tumors compared to metastases (34). However, no validation of these markers was reported in the study population. In a follow-up of primary SI-NET and liver metastases, it was selected a panel of epigenetically dysregulated genes that were progressively methylated or demethylated from the primary tumor to metastases (33, 41), suggesting their potential use as markers. Recently dysregulation of TET1/TET2 enzymes that catalyze DNA demethylation was observed in SI-NETs open a potential novel class of drug treatment (42, 43). Differential methylations of specific gene promoters were also associated with response to therapy. **Table 1** shows the most representative observational studies involving epigenetic biomarkers. One example is O6-methylguanine-DNA methyltransferase (MGMT), a DNA repair enzyme removing alkyl groups from an alkylguanine. Retrospective studies have found an association between methylation of MGMT and response to treatment with temozolomide (an alkylating agent) making it a promising marker (44–47) (**Table 1**). A prospective trial confirmed this correlation (48).

**Table 1:** Observational studies on epigenomics and NETs.

Type of intervention	Drugs and targets	Phase	NIH Clinical Trialcode	End points	n. Patients
Use of Blood Biomarkers to Predict Gastric Cancer Risk	blood-based biomarkers analyses	observational	NCT04329299 (2012-2016)	Micro RNAs (miRNAs) and blood-based protein markers in participants	6,862
Tissue Procurement for Gastric Cancer including neuro endocrine cancer,	blood-tissues from any kind of treatment	observational	NCT01416714 (2011-2025)	Blood -tissues collection	1,000
A Collection of Clinical and Epidemiologic Data Combined With Tissue and Blood From Patients With a Diagnosis of Neuroendocrine Tumors GEPNET or NET of unknown primary.	blood- and biopsies collection from any kind of treatment	observational	NCT00745381 (2008-2020)	Blood-based biomarkers molecular testing include evaluation of DNA mutation, alternative splice variants, protein expression and phosphorylation, and immunohistochemistry on sample analyses	500
Community-based Neuroendocrine Tumor (NET) Research Study	Drug: lanreotide target: somatostatin receptor	observational	NCT02730104 (2015-2020)	Data collected will be in accordance with the routine practice of physicians. Blood collection	100
Database ITANET - ENETS Registry	all treatment	observational	NCT04282083 (2020-2022)	Create an Italian database for the collection of data on diagnostic approach, therapy and follow up of patients affected by GEP-NET (gastro-enteric-pancreatic neuroendocrine tumors) and to include these data into a multi-national European registry database, adhering to the ENETS (european neuroendocrine tumor society)-registry project.	3,600
Integrated Cancer Repository for Cancer Research (iCaRe2) including neuro endocrine	all treatments	observational	NCT02012699 (2013-2099)	Register: observational, genetics, biology, early detection, and patient care can collaborate by using the iCaRe2 as a platform for cohort and population studies.	999
SYNERGY-AI: Artificial Intelligence Based Precision Oncology Clinical Trial Matching and Registry	all treatments	observational	NCT03452774 (2018-2021)	Platforms, individual clinical data is extracted, analyzed and matched to a parametric database of existing institutional and non-institutional CT.	1,500
The Lyon Real World Evidence in Metastatic NeuroEndocrine Tumours (LyREMeNet)	advance stage	observational	NCT03863106 (2017-2020)	Clinical characteristics, prognostic factors, treatment patterns, and the overall survival among patients with metastatic GEP and lung NETs.	880
Treatment With Somatostatin Analogues in Patients With Gastroenteropancreatic Neuroendocrine Tumours (STREET)	Drug: somatostatine, targe:t receptor for somatostatine	observational	NCT02788565 (2016-2020)	Direct Cost of Treatment and quality life	156
A Safety and Tolerability Study of INCAGN02390 in Select Advanced Malignancies	all treatments	observational	NCT04028479 (2019-2021)	Diagnostic Test: Testing: Genome Diagnostic Test: Testing: Transcriptome Diagnostic Test: Testing: Proteome Drug: Treatment: CAR-T	10,000
The PIONEER Initiative: Precision Insights On N-of-1 Ex Vivo Effectiveness Research Based on Individual Tumor Ownership (Precision Oncology) (PIONEER)	with or without therapy	observational	NCT03896958 (2019-2021)	Goal of PIONEER is to enable best in class functional precision testing of a patient’s tumor tissue to help guide optimal therapy (to date this type of analysis includes organoid drug screening approache	200
Recurrence Rates of Type I Gastric Neuroendocrine Tumors Treated with Long-acting Somatostatin Analogs	Drug: octreotide analog of somatostatine	observational	NCT03812939 (2019-2020)	Clinical symtoms evaluation of miRNAs as, prognostic factors, and treatment patterns	30

## miRNAs Relevance in Differential Diagnosis and Prognosis

MicroRNAs (miRNAs) are small (19–24 nt) regulatory RNA molecules that can also be used to classify cancer because of their abundance, cell-type, and disease-stage specificity which support their possible use to predict clinical outcomes and differential diagnosis. Multiple miRNA profiling studies have been performed on NET pathological types using different RNA isolation, detection, and analysis methods. Although these differences complicate inter-study comparisons, miRNAs still hold much promise as markers. A set of 10 miRNAs (miR-99a, 99b, 100, 125a, 125b-1, 125b-2, 129-2, 130a, 132, and 342) was selected as a potential tool to differentiate pancreatic NEN from pancreatic acinar cell carcinoma (49), while miR-21a was selected as potential biomarker for GEP-NETs (50). Moreover, in another study in insulinomas, miR-204 was the unique miRNA selectively overexpressed while miR-186 showed significantly downregulated in 39 colorectal NET patients (51).

Different sets of miRNAs were identified as predictors of metastases on the base of tissue used as control. Overexpression of miR-21, involved in the regulation of the PI3K/Akt/mTOR pathway, and the Ki-67 proliferation index was significantly associated with liver metastases when pancreatic normal tissue was used as control (52). In contrast proliferation index Ki-67, miR-642, and miR-210 were correlated with metastases of PNETs when pancreatic islets were used as control (49). These data suggest that reference tissue influences the selection of markers. From the comparison of primary tumor and metastasis and then validation in 37 patients, the miRNA-196a was found significantly associated with tumor grade and recurrence (53).

A different approach is the NETest algorithm for the prediction of the clinical status of NETs (54, 55). The test is PCR-based measuring 51 individual circulating genes in 1 ml of blood. An algorithmic analysis provides a numeric score of disease status. It can define the completeness of surgical resection, identify residual disease, monitor disease progression, and determine the efficacy of treatment (56–58). NETest was used to evaluate the alteration in genes during the treatment with SSA, PRRT, and following surgery (59–61). In a Dutch cohort of GEP-NET patients, the NETest had good sensitivity but the specificity was relatively low. Thus, NETest would be less suited for screening but could be valuable for the detection of residual disease after therapy (62). Interestingly, the NETtest was successfully used to evaluate efficacy and response to PRRT in metastatic NETs (63) (**Table 1**).

Several miRNAs were also associated with tumor progression of SI-NETs. In the miRNAs study performed by Heverhagen (64), the most promising diagnostic miRNA-biomarker was miR-7-5p higher in pathological tissue compared to control and selected miR-885-5p as predictive of rectal NETs metastases (65). In a cohort study, 3 miRNAs 129-5p, 133a, and 143-3p downregulated were associated with the metastatic phenotype of SI-NETs (64, 66–70), while other upregulated were correlated with SSA treatment status or tumor stage (71). In another study, 4 differentially expressed miRNAs (miR-21-5p, miR-22-3p, and miR-150-5p) reached a statistical significance (72) underlying the need to add tissue markers, to discriminate NETs and to confirm the findings in annotated sample sets. Two miRNA profiling studies conducted on SI-NETs (66, 69, 73), compared metastatic tumors to primary malignancy, merging the data from both studies (metastasis vs primary) downregulation of miR-133a and upregulation of miR-183 were associated with poor prognosis and the spread of malignancy.

## Role of Long Non-Coding RNAs in NENs Clinical Findings

Long non-coding RNAs (lncRNAs) are non-protein coding RNA transcripts longer than 200 nucleotides that exert multiple types of regulatory functions of all known cellular processes. Increasing evidence supports the role of lncRNAs in NENs development and progression with different mechanisms. In PNETs, tumor hypermethylation and silencing of long noncoding MEG3, determined activation of miR183/BRI3 axis, and cell proliferation due to c-MET oncogene activation (73). The reactivation of MEG3 by demethylating agents suppresses c-MET dependent cell proliferation suggesting that epigenetic targeting of MEG3 may represent an interesting approach in MEN1-PNETs treatment (59).

Moreover, downregulation of noncoding MEG3 and HOX genes has been associated with the development of non-functional pituitary adenomas and parathyroid tumors, respectively (74).

Two other lncRNAs are implicated in the pathogenesis of PNENs, the HOX antisense intergenic RNA chromatin-modifier (HOTAIR) and the metastasis-associated lung adenocarcinoma transcript 1 (MALAT1) (75). HOTAIR reprograms neuroendocrine differentiation of prostate cancer (76), and its overexpression increases H3K27me and metastatic potential of breast cancer cells (77). Evidence supports the hypothesis that both lncRNAs through epigenetic modification activate downstream pathways Wnt/β-catenin (78) and ERK/MAPK (79) promoting epithelial-mesenchymal transition (EMT). In contrast, the upregulation of both lncRNAs in primary GEP-NETs was associated with less aggressive disease (80), as well as lncRNA, telomeric repeat-containing RNA (TERRA), is necessary to maintain genome integrity (81).

## Epigenetic Modifications Assessed in Liquid Biopsies as Prognostic Markers

Unlike traditional tissue biopsies, liquid biopsies are faster, less invasive, have the potential to reflect all metastatic sites (i.e. tumor heterogeneity), and can indicate therapeutic response or progression through serial sampling. By considering the potential of genomic analysis, liquid biopsies offer a facilitated means of detecting genomic alterations and can be easily repeated over time. Moreover, cancer-specific circulating DNA (ctDNA) methylation can be used to measure circulating tumor DNA, as well as reveal the methylation patterns in the tumor (10).

In metastatic PNET patients, free circulating DNA carrying oncogenic mutations or methylation have been identified by mutation-specific droplet digital PCR (ddPCR) (82). In particular in a prospective trial (“MGMT-NET”), MGMT hypermethylation was also detectable in ctDNA instead of tissue (83, 84).

The Phase II PAZONET study is evaluating the epigenome modification in circulating tumor cells (CTCs), as potential biomarkers of response to therapy. The same goal was also assessed during SSA treatment in association with PRRT (85–87). This novel approach indicates that epigenetic profiling can identify serum biomarkers with prognostic potential (10).

## Epigenetic Targeted Agents and Clinical Trials

**Figure 1:**
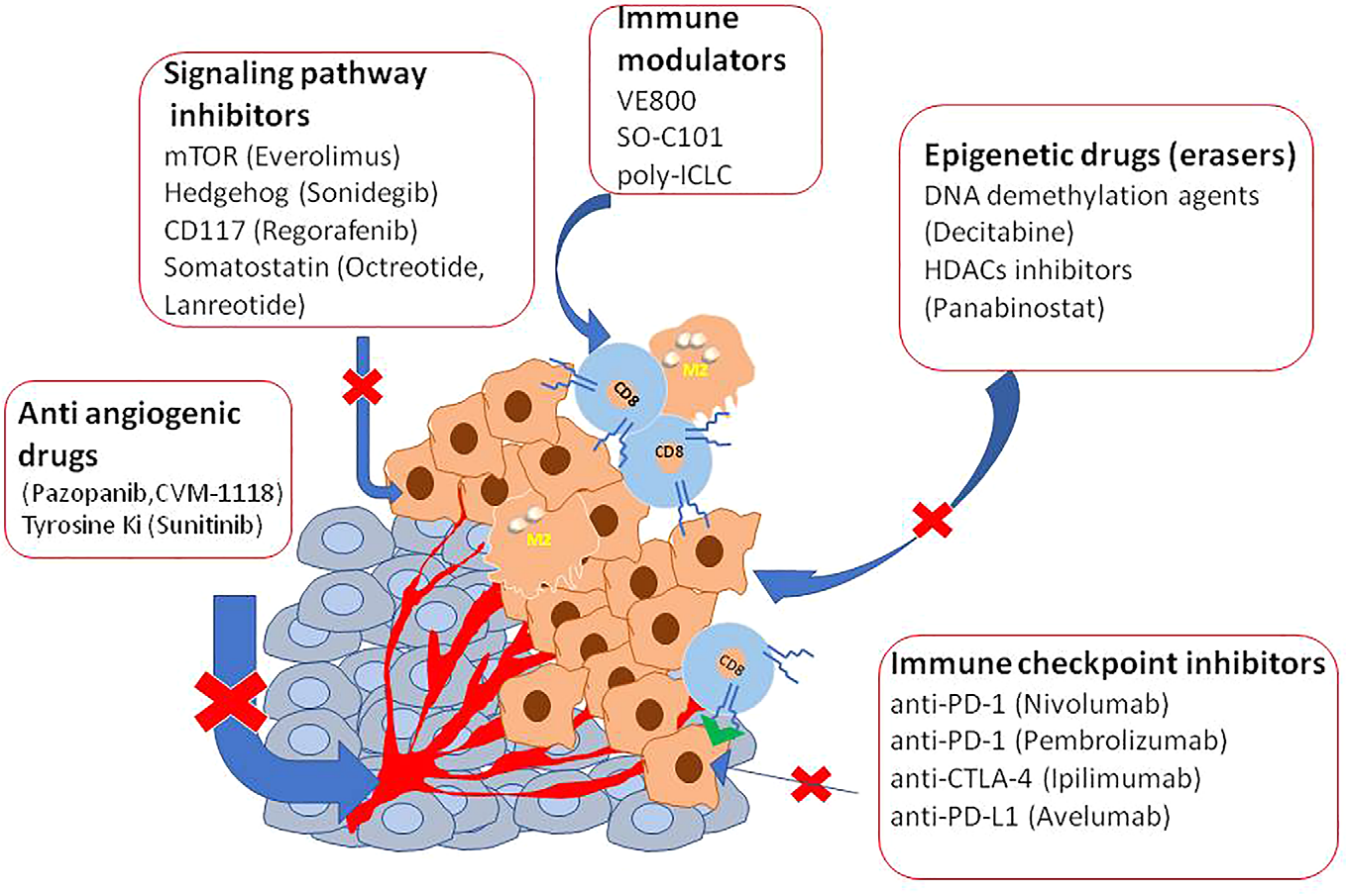
A list of epigenetic agents useful in the therapy of NETs.

Several clinical studies reported disease control targeting the somatostatin receptor (SSR), overexpressed in 70% of GEP-NETs, supporting the efficacy of both the available SSA octreotide and lanreotid) (88–90). To improve the efficacy and adverse metastatic phenotype, several ongoing trials are evaluating other targets as an inhibitor of angiogenesis, immunotherapy, or combinations of them (**Table 2** and **Figure 1**).

**Table 2:** Clinical trials with drugs interfering with epigenetic pathways.

Hystology	Drugs and targets	Phase	NIH Clinical Trial	End points	n. Patients
**Monotherapy**					
Solid tumor including adenocarcinoma gastric cancer	Drug: MLN8237 target **aurora kinase**	Phase I/II	EudraCT: 2008-006981-27 (2011 completed)	Safety, tolerability, and efficacy	273
Advanced Neuroendocrine Cancer	Drug: pazopanib target **antiangiogenesis**	Phase II	NCT00454363 (2007-2015)	Disease progression laboratory biomarker	52
Low grade neuroendocrine tumor	Drug: panabinostat target **HDACis**	Phase II	NCT00985946 (2010-2015)	Response to therapy	15
Gastro-enteropancreatic metastatic Neuroendocrine Tumor	Drug: Famitinib target **c-Kit, VEGFR2, PDGFR, VEGFR3, Flt1 and Flt3**	Phase II	NCT01994213 (2015-2019)	Efficacy and molecular testing include evaluation of DNA mutation, and immunohistochemistry	53
First-line treatment in newly-diagnosed patients with Advanced GI Neuroendocrine Tumors.	Drug everolimus, target: **mTor**	Phase II multicenter	NCT01648465 (2012-2019)	Efficacy as first line	25
Gastroenteropancreatic Neuroendocrine Tumor G3	Drug: Anlotinib target: **tyrosin kinase inhibitor** VEGFR2, PDGFR, VEGFR3, Flt1 and Flt3	Phase II	NCT03457844 (2018-2019)	Clinical and molecular data of disease progression	60
A Safety and Tolerability Study of INCAGN02385 in Select Advanced Malignancies	Biological: INCAGN02385 target **LAG3**	Phase I	NCT03538028 (2018-2020)	Safety	40
In Patients With Advanced Neuroendocrine Tumors After Progression on Everolimus (CABINET)	Cabozantinib S-malate, target: **inhibtor tyrosine kinase** VEGFR2 RET MET AXL	Phase III randomized	NCT03375320 (2017-2021) (2018-2021)	Efficacy as first line	395
Treatment of Advanced Adult Solid Tumors including gastric and neuroendocrine	Drug: VMD-928Capsules target **tyrosine kinase**	Phase I	NCT03556228 (2018-2021)	Safety	54
Select Advanced Malignancies and Neuroendocrine Tumor	Drug: INCAGN02390 target: **antagonize the TIM-3 pathway**	Phase I	NCT03652077 (2019-2021)	Safety	41
Refractory Solid Tumors, Esophageal Carcinoma Gastric (The MATCH Screening Trial)	Drug: Crizotinib **Inhibitor of ALK and ROS1**	Phase II	NCT02465060 (2015-2022)	End-of-treatment biopsy and collection of blood samples for research purposes	6,245
Study of CVM-1118 for Patients With Advanced Neuroendocrine Tumors	Drug: CVM-1118 inhibitor of **vasculogenic mimicry**	Phase II	NCT03600233 (2018- 2022)	Efficacy	30
Unresectable Gastroenteropancreatic Neuroendocrine Tumors (GEP NETs)	Drug: Abemaciclib, target: CDK4/cdk6 inhibitors	Phase II	NCT03891784 (2019-2024)	Disease progression	37
Patients With Grade 2 and Grade 3 Advanced GEP-NET (NETTER-2)	Drug: Lutathera Drug: long-acting octreotide target **receptor somatostatin**	Phase III Multi-center, Randomized	NCT03972488 (2020-2026)	Efficacy of treatment	222
Observational Study Following Neuroendocrine tumor	FT500 **Cellular Immunotherapy** Allogeneic natural killer (NK) cells	Phase I observational	NCT04106167 (2019-2034)	Safety	76
Malignant Esophagogastric Neoplasm MAGE-A4^c^¹°³²T for Multi-Tumor	Autologous genetically modified MAGE-A4^c^¹°³²T cells in subjects who have the appropriate HLA-A2 tissue marker	Phase I	NCT03132922 (2017-2035)	Safety	42
Gastric cancer	Drugs: Atezolizumab target: **PD-L1 immune-checkpoint**	Phase II	EudraCT:2015-000269-30 (2015-ongoing)	Tolerability and efficacy	725
Pancreatic neuroendocrine tumor	Drug: sunitinib target: **tyrosine kinases**	Phase II	EudraCT: 2012-000425-45 (2012-ongoing)	Effects of morning vs evening dosing on the pharmacokinetics and pharmacodynamics of sunitinib	18
**Combination therapy**					
Patients with unresectable, Neuroendocrine Tumor Metastatic Liver Cancer	Drug: cyclophosphamid **chemotherapy** Drug: poly-ICLC **immuno-stimulatory agent** Radiation	Phase I/II	NCT00553683 (2007-2014)	Safety and efficacy	50
Advanced Metastatic NETs (COOPERATE-1)	Pasireotide target: **somatostatin receptor** Drug: Everolimus Targets: **mTor**	Phase I	NCT01263353 (2010-2016)	Safety	36
Gastroenteropancreatic Neuroendocrine Tumors (PLANET)	Drug: Somatuline Depot target **receptor somatostatin** Drug: Keytruda anti (PD-1) **immune-checkpoint**	PhaseI/II	NCT03043664 (2017-2020)	Clinical and molecular data of disease progression	22
Gastro-enteropancreatic Neuroendocrine Tumor (REGOMUNE)	Drug: Regorafenib targeting **cKIT** Drug: Avelumab anti PD-L1 **immune-checkpoint**	PhaseI/II	NCT03475953 (2018-2021)	Disease progression and efficacy	362
Advanced Solid Tumors (DUET-2) Neuroendocrine Tumor	Drug: XmAb20717 target: **bispecific antibody** anti PD-L1/CTLA-4	Phase I	NCT03517488 (2018-2021)	Safety	154
Advanced Gastrointestinal Neuroendocrine Tumor G3 Type	Drug: Etoposide **chemotherapy** Drug: Irinotecan target **inhibitor of topoisomerase** I.	Phase II	NCT03963193 (2019-2021)	Disease progression	100
Patients With Advanced Solid Tumors and Neuroendocrine Tumor	Drug Pembrolizumab, **target: PD-1 immune-checkpoint** Drug: Sonidegib target: **Hedgehog signaling** PD1 pathway	Phase I	NCT04007744 (2019-2021)	Safety	78
Advanced or Metastatic Cancer (Consortium-IO) Esophageal Cancer Neuroendocrine Tumor	Drug: Nivolumab optidivo **immune-checkpoint** Drug: Vancomycin targeting VE800 **activates CD8 cells**	Pase I/II	NCT04208958 (2019-2022)	Safety and clinical activity	111
Advanced Solid Tumors And neuroendocrine tumor	Drug: **FT500** Drug: **Nivolumab** **Drug: Pembrolizumab** Drug: Atezolizumab Drug: Cyclophosphamide Drug: Fludarabine	Phase I	NCT03841110 (2019-2022)	immunotherapy tolerability	76
Advanced/Metastatic Solid Tumors Gastric Cancer Neuroendocrine Tumor	Drug: SO-C101 target: **super agonist IL15**; Drug: pembrolizumab anti **PD-1**	Phase I	NCT04234113 (2019-2022)	Safety	96
A Study to Evaluate the Safety and Pharmacokinetics of OC-001 in Patients With Locally Advanced or Metastatic Cancers including Neuroendocrine Tumor	Drug: OC-**001** target: **TNF,** Drug: **pembrolizumab anti PD-1**	Phase I/II	NCT04260802 (2020-2022)	Efficacy	80
Locally Advanced or Metastatic Solid Tumors including gastric neuro endocrine	Drug: INBRX-106 - Hexavalent OX40 **agonist antibody TNF** Drug: **Pembrolizumab anti PD-1**	Phase I	NCT04198766 (2019-2023)	Safety	150
Advanced or metastatic cervical cancer, endometrial cancer, gastric cancer, hepatocellular carcinoma	Drug: **INCAGN01876** inhbitor of LAG3 Immune Therapies **PD-1/PD-L1 therapy**	Phase I/II	EudraCT 2016-004989-25 (2017-ongoing)	Safety, Tolerability, and Efficacy	
Gastric Cancer (GC) Pancreatic Cancer (PC)	Drugs: Nivolumab target **PD-1** 1 and Ipilimumab target: **CTLA-4**	Phase I/II	EudraCT: 002844-10 (2013-ongoing)	Safety, Tolerability, and Efficacy	

Targets are in bold.

Epigenetics represents a very promising tool in cancer treatment because it can be reverted and epigenetic drugs are in use for the treatment of several cancer types (10, 91).

In vitro studies have already tested DNA methyltransferase inhibitors (DNMTis) because of MEN1 loss increase DNA hypermethylation (92). Promising results in PNET and small intestine cell lines were obtained using inhibitors of DNA methylases and HDAC to reduce cell viability and restoring gene expression (93–97). Interestingly, decitabine increased the expression of SSTR2 and the Ga-DOTATOC uptake also in BON1 tumor-bearing mice, indicating a possible therapy implication (98). However, decitabine has not yet been trialed in humans mainly because this agent targeted the whole methylated genome. Panobinostat, a histone deacetylase inhibitor (HDACi), was used in a completed phase II trial for the treatment of low-grade NENs. Patients showed a high stable disease with the median progression-free survival (PFS) of 9.9 months, and the median overall survival was 47.3 months. However, the low response rates, limitated further investigation (99). Inhibitors of the Bromo and extra terminal domain (BET) protein family, epigenetic readers of histone code, have also tested in experimental models (100). Of particular interest is Rx-001 which acts by blocking both DNMT and HDACs, activity. It showed to induce global epigenetic changes in tumors favoring infiltration of T cells, this histology was correlated with clinical benefit and sensitize tumor microenvironment to chemotherapy (101).

Novel frontier in solid tumor treatment is evaluating a combination of immunotherapy with epigenetic drugs, mainly because some immunosuppressive cancer antigens are regulated by acetylation of their genomic regulative element.

Some trials are testing a combination of agonists of TNF and immunotherapy via checkpoint inhibition (NCT04198766) or antibody with double specificities against PD-L1 and CTLA-4 (NCT03517488). However major interest gained depleting tryptophan enzymes as indoleamine 2,3-dioxygenase and tryptophan 2,3-dioxygenase (ICI). This because tryptophan is able to induce immune suppression within the cancer microenvironment. In tumor cells and nude mice have already targeted tryptophan. The authors by specific inhibitors or by preventing tryptophan promoter acetylation using histone deacetylase inhibitors as BET reported the reduction of immunosuppressive protein expression (10, 102) suggesting a novel therapeutic approach.

## Conclusions and the Way Forward

**Figure 2:**
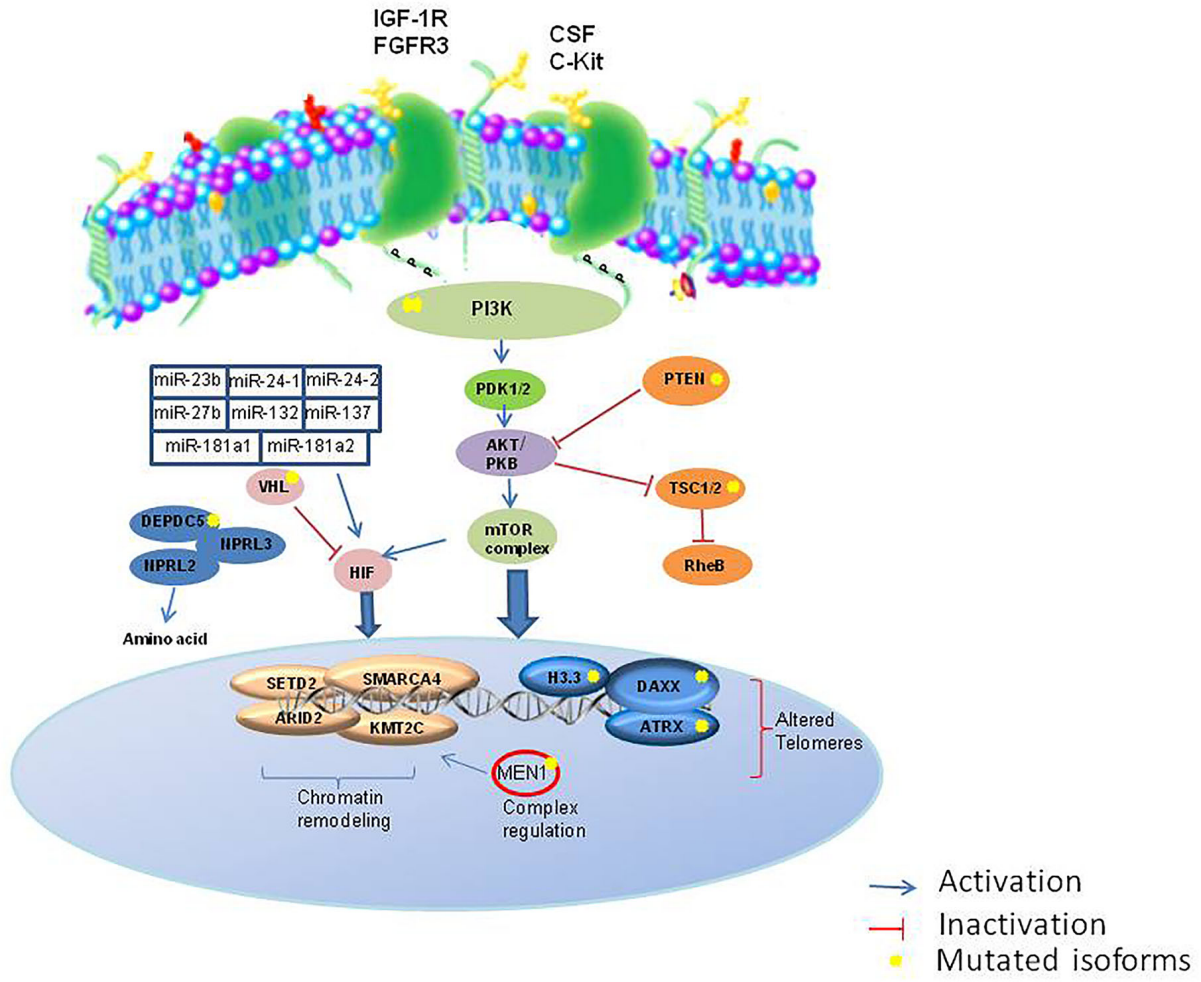
Major epigenetic pathways involved in NETs. IGF1R, insulin growth factor 1 receptor; FGFR, fibroblast growth factor receptor; SCF, colony stimulation factor; c-KIT, c-Kit proto-oncogene; PI3K, phosphatidylinositol 3-kinases; PTEN, Phosphatase and tensin homolog; PDK1/2, protein 3-phosphoinositide-dependent protein kinase-1; TSC1/2, Tuberous sclerosis 1/2; RheB, Ras homolog enriched in brain; HIF, hypoxia factor; RheB, Ras homolog enriched in brain; VHL, Von Hippel-Lindau; DEPDC5, DEP domain containing 5; NPRL3, neuropilin 3.

The development of high-throughput techniques and larger datasets (i.e. The Cancer Genome Atlas) have accelerated research even in the field of NENs. Some pioneer studies have used an integrative approach in GEP-NETs (103). EWAS showed that these epigenome profiles can distinguish subtypes with different clinical features (**Figure 2**). The development of the NETest and liquid biopsy, as well as organoids (104), can be used to predict response to therapy and during the clinical follow-up, although not routinely used. Recently, it was proposed a bioresponsive drug-delivery depot for a combination of epigenetic modulation and immune checkpoint blockade (105). From the analysis of the clinical trials reported in **Table 1** and **Table 2**, it emerges that the evaluation of the epigenetic pathway as a biomarker of response is of most interest in many studies, involving different kinds of therapies, even in combination (10). NCT03475953 and NCT03841110 ongoing trials are evaluating the therapeutic potential of the combination of direct drugs against tyrosine kinases and immune response pathways such as PD-1/PD-L1 and the opportunity to select from patients’ blood epigenetic biomarkers. The major challenge will now be to clinically validate such epigenetic biomarkers, within clinical trials for therapeutics in the new light of precision medicine, as well as network medicine (104, 106–108).

## Author Contributions

AC, FN, and CN contributed to conception and design of the study. FN, RM and CN analyzed the data and wrote sections of the manuscript. All authors contributed to the article and approved the submitted version.

## Funding

This work was funded by POR Campania FESR 2014-2020 “RARE.PLAT.NET” CUP B63D18000380007. This work was supported in part by “Ricerca Finalizzata 2011–12” (project code RF-2011-02349443, PI CN).

## Conflict of Interest

The authors declare that the research was conducted in the absence of any commercial or financial relationships that could be construed as a potential conflict of interest.

## Abbreviations

CAPTEM, Capecitabine and temozolomide; cfDNA, Cell-free DNA; CIMP, CpG island methylator phenotype; CNV, Copy number variation; CTC, Circulating tumor cell; ctDNA, Circulating tumor DNA; ddPCR, Droplet digital PCR; GEP, Gastroenteropancreatic; MEN1, Multiple endocrine neoplasia type 1; NEN, Neuroendocrine neoplasm; NET, Neuroendocrine tumor; NEC, Neuroendocrine carcinoma; NF1, Neurofibromatosis type 1; PNEC, Pancreatic neuroendocrine carcinoma; PNEN, Pancreatic neuroendocrine neoplasm; PNET, Pancreatic neuroendocrine tumor; SI-NET, small intestinal neuroendocrine tumors; VHL, Von Hippel Lindau.
